# Estimated incubation period for monkeypox cases confirmed in the Netherlands, May 2022

**DOI:** 10.2807/1560-7917.ES.2022.27.24.2200448

**Published:** 2022-06-16

**Authors:** Fuminari Miura, Catharina Else van Ewijk, Jantien A. Backer, Maria Xiridou, Eelco Franz, Eline Op de Coul, Diederik Brandwagt, Brigitte van Cleef, Gini van Rijckevorsel, Corien Swaan, Susan van den Hof, Jacco Wallinga

**Affiliations:** 1 Centre for Infectious Disease Control, National Institute for Public Health and the Environment (RIVM), Bilthoven, the Netherlands; 2 Center for Marine Environmental Studies (CMES), Ehime University, Ehime, Japan; 3 European Programme for Intervention Epidemiology Training (EPIET), European Centre for Disease Prevention and Control (ECDC), Stockholm, Sweden; 4 Department of Infectious Diseases, Public Health Service Amsterdam, Amsterdam, the Netherlands; 5 Department of Biomedical Data Sciences, Leiden University Medical Center (LUMC), Leiden, the Netherlands

**Keywords:** Monkeypox, incubation period, symptom onset, smallpox, varicella virus infection (chickenpox), poxvirus, quarantine, epidemiology, modelling, statistics

## Abstract

In May 2022, monkeypox outbreaks have been reported in countries not endemic for monkeypox. We estimated the monkeypox incubation period, using reported exposure and symptom-onset times for 18 cases detected and confirmed in the Netherlands up to 31 May 2022. Mean incubation period was 8.5 days (5th–95th percentiles: 4.2–17.3), underpinning the current recommendation to monitor or isolate/quarantine case contacts for 21 days. However, as the incubation period may differ between different transmission routes, further epidemiological investigations are needed.

Since the beginning of May 2022, monkeypox outbreaks have been reported in countries, predominantly in Europe, where the monkeypox virus is not endemic [1]. Key public health measures to stop the spread of infection include active case finding, contact tracing, and isolation or quarantine of close contacts. The incubation period of monkeypox has been reported to be up to 21 days, prompting public health institutes to recommend active monitoring and isolation/quarantine of close contacts for a minimum of 21 days after the last day of exposure [1-3].

The duration of the incubation period for monkeypox is known to depend on the transmission route [4]. It is therefore essential to establish the distribution of the incubation period in the recent outbreaks. Whereas cases in previous outbreaks of monkeypox in non-endemic areas had travelled to endemic countries or had contact with infected animals [5], the 2022 outbreaks [1] affect many cases with no documented history of travel to endemic countries and who identify as men who have sex with men (MSM) [6,7]. Close contact during sexual activity may play an important role in transmission during the current outbreaks. Given the particular types of exposures and differences in route of transmission, the incubation period for monkeypox in the current outbreaks may also have a different duration.

Here we estimate the incubation period of monkeypox using the reported time of exposure and symptom onset for confirmed monkeypox cases recently detected in the Netherlands up to 31 May 2022.

## Observed incubation periods

In the Netherlands, monkeypox was classified as a group A notifiable disease on 21 May 2022. This means that suspected and confirmed cases of monkeypox should be immediately notified to the public health services. As of 31 May, 31 monkeypox cases were laboratory-confirmed by PCR in the country. All cases were men and identified themselves as MSM, and the age range was 23–64 years old. At data collection, 18 cases had reported the symptom onset date and the most likely date of exposure as a single date or a limited number of consecutive dates, related to the attendance of an event where exposure was considered most likely.

We fitted parametric distributions to the observed incubation periods among 18 cases with symptom onset and exposure histories for monkeypox, using a likelihood-based approach, which allows for exposure to be a single time point or a time interval [8]. The computation was implemented in R-4.0.5 [9] with a package {rstan}-2.21.2 [10]. We compared three alternative parametric distributions: the lognormal, the gamma, and the Weibull distribution, and selected the best fitting distribution.

The reported incubation intervals for monkeypox were best described by a lognormal distribution (Table 1). Using this best-fitting distribution, the mean incubation period was estimated to be 8.5 days (95% credible intervals (CrI): 6.6–10.9 days), with the 5th percentile of 4.2 days and the 95th percentile of 17.3 days (Table 2).

**Table 1:** Estimated mean of monkeypox incubation period according to different parametric distributions and computed goodness-of-fit, the Netherlands, May 2022 (n = 18 cases)

Distribution	Mean in days (95%CrI)	WAIC	LOOIC^a^
Lognormal	8.5 (6.6–10.9)	99.8	100.7
Gamma	9.1 (7.5–11.3)	102.1	103.0
Weibull	9.6 (7.4–12.4)	104.6	105.2

CrI: credible interval; LOOIC: leave-one-out information criterion; WAIC: widely applicable information criterion.

^a ^These values indicate the goodness-of-fit, where lower values indicate a better fit.

**Table 2:** Estimated percentiles of the incubation period for monkeypox, using different parametric distributions, the Netherlands, May 2022 (n = 18 cases)

Percentile	Lognormal		Gamma		Weibull	
	Estimate	95% CrI	Estimate	95% CrI	Estimate	95% CrI
2.5^th^	3.6	2.0–5.0	3.8	2.0–5.2	2.3	0.9–4.1
5^th^	4.2	2.5–5.5	4.4	2.6–5.8	3.1	1.4–5.0
50^th^	8.5	6.6–10.9	8.7	7.0–10.7	9.2	6.9–11.8
95^th^	17.3	13.0–29.0	15.3	12.5–20.7	16.9	13.7–23.9
97.5^th^	19.9	14.4–35.7	16.9	13.6–23.3	18.5	14.9–26.9
99^th^	23.3	16.3–45.8	18.8	14.9–26.7	20.3	16.1–30.6

CrI: credible interval.

Visual inspection revealed a good match between the fitted cumulative lognormal distribution function and the empirical cumulative distribution function, including the right tail of the distribution that describes the frequency of long incubation periods (Figure). The 2.5 percentile for the incubation period is estimated to be 3.6 days, and the 97.5 percentile is estimated to be 19.9 days (Table 2). An estimated two per cent of all cases would develop first symptoms more than 21 days after being exposed.

**Figure:**
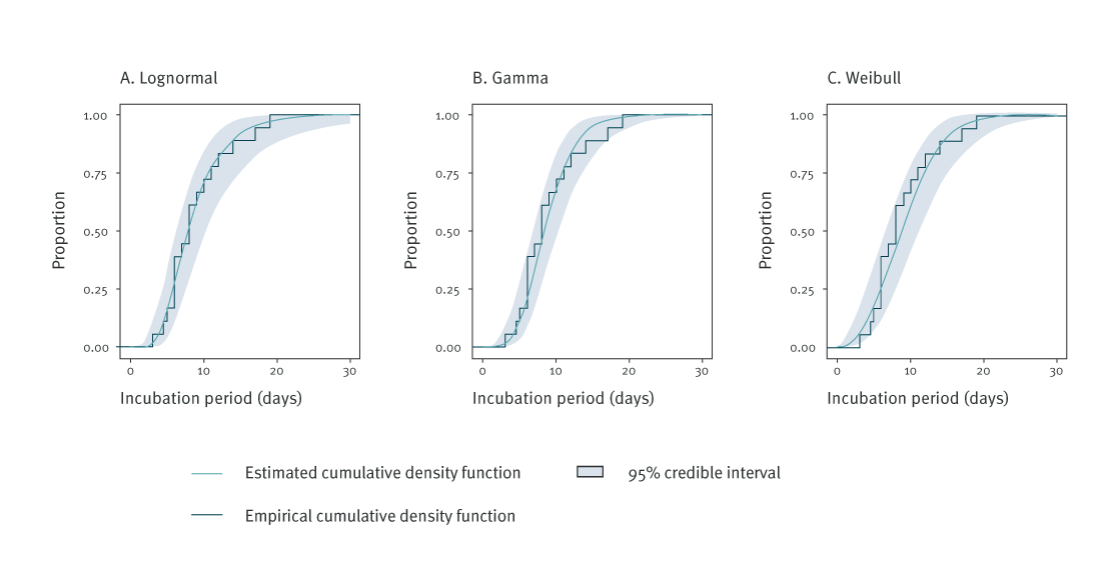
Estimated cumulative density functions, according to different parametric distributions and empirical cumulative density function of incubation periods reported for monkeypox cases in the Netherlands, May 2022 (n = 18 cases)

For the visualisation of the empirical cumulative distribution, the median points of the reported time interval of exposure were used.

## Discussion

This study provides empirical evidence for the distribution of the incubation period, using the data on exposure histories and symptom onsets of monkeypox cases recently laboratory-confirmed by PCR in the Netherlands up to 31 May 2022. The estimated 95th percentile of 17.3 days and the 97.5th percentile of 19.9 days can underpin the usage of 21 days for monitoring or isolating/quarantining close contacts of cases to limit further spread of the infection.

The duration of the incubation period has been reported to differ by route of transmission for monkeypox virus, smallpox, and vaccinia viruses [4]. For non-invasive exposure (e.g. intact skin contact or droplet transmission) the typical incubation period of monkeypox is 13 days, and for complex and invasive exposures (e.g. contact with broken skin or mucous membranes), the typical incubation period is 9 days [4]. These values are consistent with those of smallpox: ca 12 days for outbreaks where exposure is predominantly non-invasive [11,12] and ca 9 days for inoculation where exposure is invasive [13]. Our estimate of the mean incubation period of monkeypox in this outbreak of 8.5 days in line with the typical values for complex, invasive exposure. This result is supported by the epidemiological observation that all notified cases currently reported in the Netherlands are MSM, mostly with lesions in the anal and genital regions. Direct contact between respective broken skin or mucous membranes during sexual activity might be the most likely route of transmission among cases reported in the current outbreak.

If the reported incubation periods are those of the first observed cases in a growing outbreak, infected persons with a long incubation period would have a lower probability to be included, relative to infected persons with a short incubation period. This could imply that the estimated incubation periods may suffer from downward bias, and that more than two per cent of all infected cases would develop first symptoms more than 21 days after being exposed. In addition, the current estimate is based on 18 confirmed cases, and thus the continued monitoring of incubation periods of cases will provide more precision as the epidemic grows. More epidemiological information on details of possible exposure routes is required to establish whether the results of the current study are generalisable to other MSM cases in the current outbreak, and to what extent the incubation period differs between alternative transmission routes.

## Conclusion

In conclusion, this report presents a plausible range of incubation periods for the 2022 monkeypox outbreaks. The estimated mean incubation period is in line with previous findings for complex, invasive exposure to monkeypox. The estimated percentage of monkeypox cases that would develop symptoms after the conclusion of 21 days period is approximately two per cent. These findings suffice for justifying the current use of 21 days for quarantining (or other approaches to avoid infectious contacts), but as the outbreaks grow and cases can be infected via different transmission routes, continued monitoring of the incubation period for monkeypox is necessary.
