# Author’s correction for Euro Surveill. 2022;27(24)

**DOI:** 10.2807/1560-7917.ES.2023.28.14.230406c

**Published:** 2023-04-06

**Authors:** Eurosurveillance editorial team

**Affiliations:** 1 European Centre for Disease Prevention and Control (ECDC), Stockholm, Sweden

In the article ‘Estimated incubation period for monkeypox cases confirmed in the Netherlands, May 2022’ by Miura et al., the mean incubation period was reported as 8.5 days, upon publication on 16 June 2022. This reported value was the median value. The actual mean incubation period is 9.0 days. This was corrected on 3 April 2023 at the request of the authors.

